# Necrotizing fasciitis after total abdominal hysterectomy: A case report

**DOI:** 10.1097/MD.0000000000034451

**Published:** 2023-08-04

**Authors:** Miloš Pantelić, Marko Sinisa Stojić, Đorđe Petrović, Ljiljana Mladenović-Segedi, Branislava Baturan, Igor Tesić, Borislav Golijan

**Affiliations:** a University of Novi Sad, Faculty of Medicine, Novi Sad, Serbia; b University Clinical Center of Vojvodina, Clinic of Obstetrics and Gynecology, Novi Sad, Serbia.

**Keywords:** case report, necrotizing fasciitis, total abdominal hysterectomy

## Abstract

Necrotizing fasciitis is rapidly progressive infection with high mortality rate. This study aimed to summarize the clinical and pathological presentation of this case.

**Patient concerns:**

A 46-year-old woman patient presented to our emergency department of an obstetric and gynecology clinic on the 8th day after total abdominal hysterectomy. The abdominal wall showed pronounced signs of inflammation. Abundant purulent content was oozing from the abdominal wound.

**Diagnoses:**

The patient underwent surgery. Areas of necrosis were observed on the skin around the wound, the subcutaneous fatty tissue was necrotic around the incision site, and the fascia was completely dehisced.

**Interventions:**

Wound debridement and flap cutting of the anterior abdominal wall were performed. Metronidazole, ceftriaxone, and vancomycin were administered intravenously. A plastic surgeon suggested daily debridement and toileting of the wound in the operating room. Swabs of the abdominal cavity, abscess cavity, and abdominal wound were obtained, and *Enterococcus faecalis* was isolated. After the negativism of microbiological swabs, excochleation of granulation tissue was performed by a plastic surgeon.

**Outcomes:**

Nineteen days after the relaparotomy, the patient was discharged in good general condition with advice for further monitoring and therapy.

**Lessons:**

Successful treatment of necrotizing fasciitis can be achieved through an initial diagnosis, adequate debridement, empirical broad-spectrum antibiotic coverage, and multidisciplinary treatment.

## 1. Introduction

Necrotizing fasciitis (NF) is a severe form of fulminating, rapidly progressive infection characterized by necrosis of the fascia, muscle, and underlying tissue. It was first coined by Wilson in 1952.^[1]^ It is a rare, but extremely life-threatening condition. Some of the early symptoms are primarily erythema, edema, fever, and pain, while late symptoms include bullaе, dysesthesia/anesthesia, hard skin upon palpation, crepitation, discoloration, and systemic manifestations. In the literature, the mortality rate is most often reported from 6% to 76%, mostly around 15% to 35%.^[2]^ Timely diagnosis and surgical resection of the affected tissue are most important in reducing the mortality rate.^[2]^

We present a case of necrotizing fasciitis of the abdominal wall in a woman who underwent a total abdominal hysterectomy.

## 2. Case report

A 46-year-old woman patient presented to our emergency department of obstetric and gynecology clinic with complaints of purulent discharge from a laparotomy wound on the 8th day after total abdominal hysterectomy.

She had a history of hypertension and asthma and had undergone appendectomy 30 years previously.

On admission, the patient was febrile (38C), pulse 90/minutes, normotensive, and hemodynamically stable. Physical examination revealed that the abdomen was respiratory-moveable and soft with no rebound tenderness or abdominal rigidity.

The abdominal wall showed pronounced signs of inflammation, was erythematous, and indurated with signs of necrosis in the middle, with dimensions of 15 × 15 mm and 40 × 25 mm. Abundant purulent content was oozing from the abdominal wound.

Under the speculum, there was an average amount of clear purulent discharge from the vagina, and the sutures on the vagina were held well.

Transvaginal ultrasound showed a neat finding.

Swabs of the vagina and abdominal wounds were taken.

**Figure 1.**
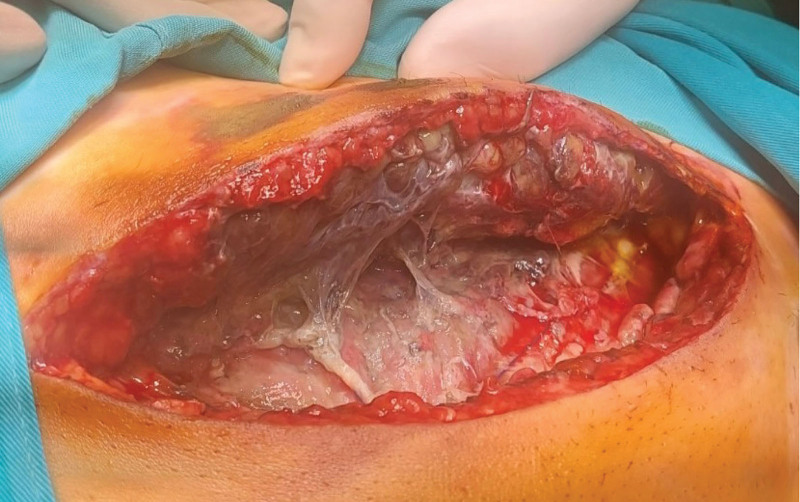
Total abdominal fascia dehiscence due to necrotizing fasciitis on the 8^th^ postoperative day after total abdominal hysterectomy.

Under short-term intravenous anesthesia, an abdominal wound was opened and a complete dehiscence of the fascia was noted (Fig. 1). Therefore, exploratory relaparotomy was indicated in the operating room.

After adequate preoperative preparation, the patient underwent surgery.

**Figure 2.**
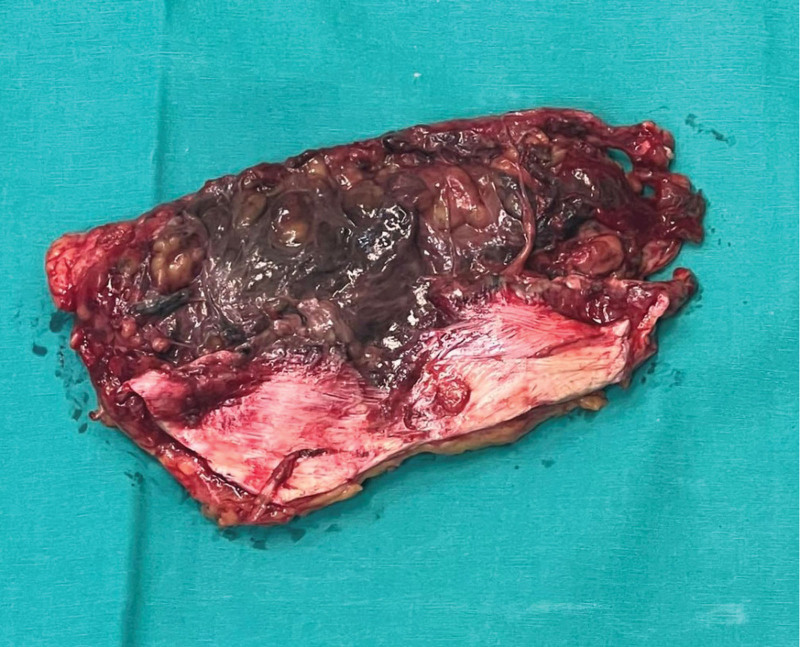
Excised part of the skin and subcutaneous tissue.

Areas of necrosis were observed on the skin around the wound, subcutaneous fatty tissue was necrotic around the incision site, and the fascia was completely dehisced. The wound on the anterior abdominal wall was debrided, and the flap od the anterior abdominal wall was cut from the proximal and distal edges of the incision (Fig. 2).

Expressed fibrin deposits were present on the muscles, and a smear was obtained from the surface of the muscles. After entering the abdominal cavity, a small abscess was observed in the region of the right adnexa, and a right-sided adnexectomy was performed. The left ovary was partially affected by inflammation, and a partial ovariectomy was performed. Owing to partial dehiscence of the sutures on the vagina, a re-suture of the vagina was performed. After washing the abdominal cavity and placing the drain, the parietal peritoneum and the muscles were sutured using individual polydioxanone II sutures. The upper and lower edges of the fascia were then brought together with 1 suture in the middle, and the wound was left to heal per second.

Laboratory findings, which were taken on admission, showed marked signs of inflammation and anemia; Leukocytes 28.59 10^9/L; Neutrophils 94.3%; C-reactive protein 171 mg/L; Hemoglobin 99 g/L; and elevated values of liver enzyme levels.

Triple antibiotic therapy was introduced, metronidazole, ceftriaxone and vancomycin intravenously.

**Figure 3.**
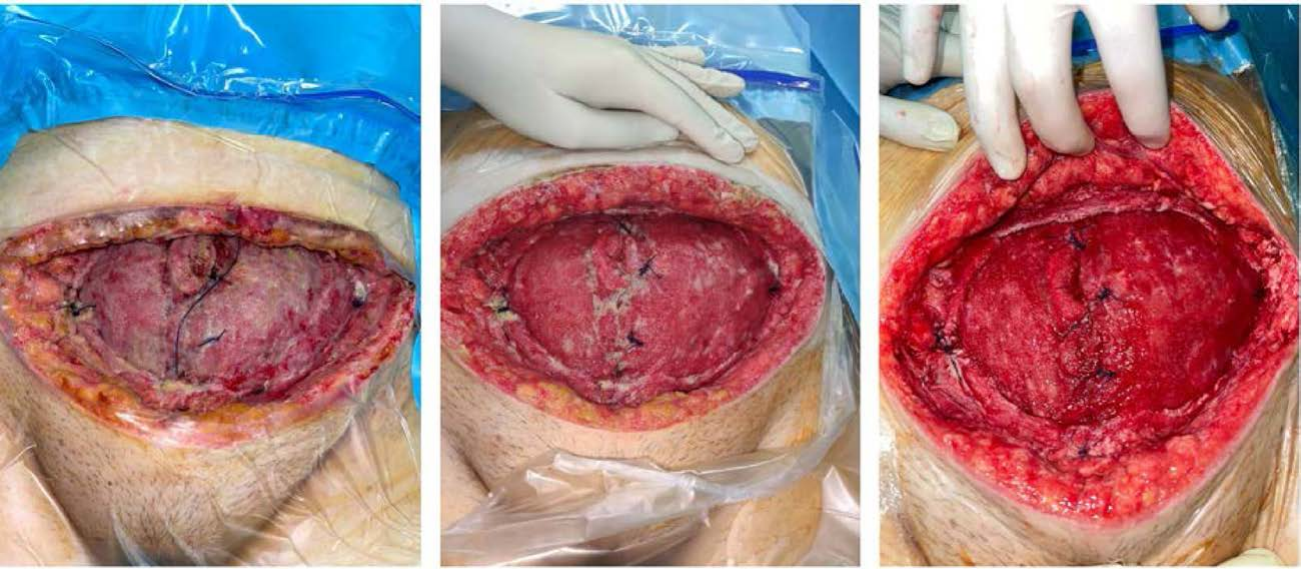
Debridement and dressing of the wound on the 12th, 16th, and 19th postoperative day.

A plastic surgeon was consulted, who suggested daily debridement and toileting of the wound in the operating room with povidone-iodine foam and solution, as well as Rivanol dressings (Fig. 3).

An infectious disease specialist was also consulted, who suggested continuation of triple antibiotic therapy.

On the second day after relaparotomy at our clinic, there was a drop in infection markers (Leukocytes 17 10^9/L, c-reactive protein 127 mg/L).

Five days after relaparotomy, enterococcus faecalis was isolated from swabs of the abdominal cavity, abscess cavity, and abdominal wound. Antibiotic therapy was prescribed according to the antibiogram (amoxicillin clavulonic acid orally).

During the postoperative course, the patient was hemodynamically stable, the markers of infection decreased, and the clinical picture improved daily.

**Figure 4.**
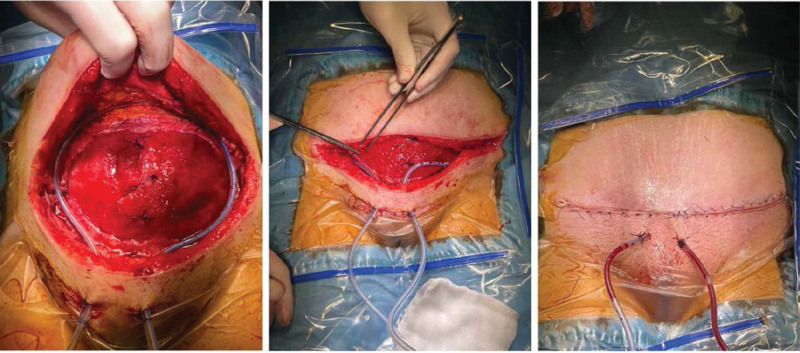
Closure and drainage of the anterior abdominal wall on the 20th postoperative day.

Twelve days after the relaparotomy (20th postoperative day), after the negativism of microbiological swabs, excochleation of granulation tissue was performed by a plastic surgeon under short-term intravenous anesthesia. The upper part of the wound was mined and 2 active drains were placed. The wound defect was closed in 2 layers of subcutaneous tissue. The skin was closed using direct sutures (Fig. 4).

Nineteen days after the relaparotomy, the patient was discharged in good general condition with advice for further monitoring and therapy. Laboratory findings were within reference values, with negative markers of inflammation. The abdominal wall was dry, clean, bandaged, and healed.

## 3. Discussion

Necrotizing soft tissue infections (NSTIs) are not only rare but also rapidly progressive and life-threatening bacterial infections that primarily cause destruction of the subcutaneous tissue and/or muscle.^[3,4]^ The incidence of NF is estimated to be 0.3 to 15 cases per 100,000 population.^[3]^ Delayed diagnosis and controversies surrounding optimal treatment represent major challenges and obstacles to improved survival.^[4]^ Patients with any skin or mucosal damage, various surgical procedures, and comorbidities such as immunosuppression, malignancy, vascular disease, diabetes, alcoholism, and obesity have an increased risk of NSTI with progression to severe sepsis and septic shock. The rapid progression of the disease and the subtlety of early signs and symptoms contribute to a high mortality rate of 25% to 35% despite increased awareness and advances in the treatment of NF and other NSTIs.^[5]^

The following classifications are based on the bacteria that initiated the cascade of injury.^[3]^ Polymicrobial (type I) infections caused by both aerobic and anaerobic bacteria account for most reported cases of NF, and are more prevalent in older adults with chronic diseases. Monomicrobial (type II) NF is most commonly associated with Gram-positive organisms such as group A Streptococcus (GAS) and methicillin-resistant *Staphylococcus aureus*.

Patients often present with signs and symptoms of infection such as erythema and edema. In order to reduce the risk of further tissue damage and death, it is important to distinguish cellulitis which can be treated with antimicrobial therapy, from necrotizing fasciitis, which requires surgical treatment, as soon as possible.^[6]^ However, as the initial clinical presentation of NF is often vague, critical diagnostic differences are usually difficult to discern. The classic symptoms of NF, such as severe pain disproportionate to the degree of apparent injury, crescendo-like pain, and fever, can be distorted by the use of nonsteroidal anti-inflammatory drugs.^[7]^ In type II NF, the patient initial superficial injury may go undetected, serious signs and symptoms may not manifest until the underlying tissue damage has advanced, and the patient is already at extreme risk of a poor outcome.^[8]^

No reliable laboratory studies have been reported for the diagnosis of NF.^[9]^ Confirmatory diagnosis of the causative bacteria is based on culture and Gram staining of samples collected from deep tissues, or positive blood cultures. A computerized tomography is preferred as the initial imaging choice because it is generally more readily available for emergent imaging.^[7]^

Clinical presentation is the most important factor in NF diagnosis. Therefore, surgical exploration is the only way to establish a diagnosis of necrotizing infection.^[10]^

Initial pharmacotherapy implies empirical administration of broad-spectrum antibiotics until Gram staining, culture, and soft tissue sensitivity results are available. The latest guidelines from the Infectious Diseases Society of America recommend either vancomycin or linezolid in combination with piperacillin-tazobactam, carbapenem, or ceftriaxone-metronidazole.^[5]^ In the present case, we were followed this recommendation and introduced ceftriaxone and metronidazole in combination with vancomycin.

Owing to its known effects on toxins released by certain organisms, including *S aureus* and GAS, clindamycin should also be included in empirical therapy. For the treatment of documented GAS necrotizing infections, penicillin plus clindamycin should be introduced.^[5]^ Once the microbiology of the specimen is determined, the clinician modifies the therapy to a specific organism using local antibiograms in order to determine the local resistance patterns, as in our patient.

When NF is suspected or diagnosed, in order to preserve viable skin and achieve hemostasis, promptly surgical removal of all necrotic tissue, including muscle, fascia, and skin is necessary, what we also proved in our case.^[11,12]^ Affected tissues should be carefully resected until only the healthy tissues remain intact. This should be continued daily, as long as the surgical team determines that all necrotic tissues have been removed. This is exactly what our team adhered to, with the help and consultation of a plastic surgeon. After aggressive surgical debridement and successful antimicrobial treatment, reconstruction using rotary flaps or skin graft techniques may be necessary; however, this was not required in our case.^[13]^

## 4. Conclusion

Successful treatment of necrotizing fasciitis can be achieved through an initial diagnosis, adequate debridement, empirical broad-spectrum antibiotic coverage, and multidisciplinary treatment.

## Author contributions

**Conceptualization:** Miloš Pantelić, Marko Sinisa Stojić, Đorđe Petrović, Branislava Baturan, Igor Tesić.`

**Data curation:** Miloš Pantelić, Marko Sinisa Stojić, Đorđe Petrović, Ljiljana Mladenović-Segedi, Branislava Baturan, Igor Tesić, Borislav Golijan.

**Formal analysis:** Miloš Pantelić, Ljiljana Mladenović-Segedi, Igor Tesić.
